# Endoplasmic reticulum stress and unfolded protein response in infection by intracellular parasites

**DOI:** 10.4155/fsoa-2017-0020

**Published:** 2017-05-12

**Authors:** Luca Galluzzi, Aurora Diotallevi, Mauro Magnani

**Affiliations:** 1 Department of Biomolecular Sciences, University of Urbino ‘Carlo Bo’, Urbino (PU), Italy

**Keywords:** *Cryptosporidium*, ER stress, immunity, *Leishmania*, *Plasmodium*, protozoan parasites, *Toxoplasma*, unfolded protein response

## Abstract

Perturbations of the physiological status of the endoplasmic reticulum (ER) trigger a specific response known as the ER stress response or unfolded protein response (UPR). In mammalian cells, the UPR is mediated by three ER transmembrane proteins (IRE1, PERK and ATF6) which activate three signaling cascades to restore ER homeostasis. In recent years, a cross-talk between UPR, inflammatory and microbial sensing pathways has been elucidated. Pathogen infection can lead to UPR activation; moreover, several pathogens subvert the UPR to promote their survival and replication. While the UPR in viral and bacterial infection has been characterized, little is known about the role of UPR in intracellular parasite infection. Here, we review recent findings on UPR induction/modulation by intracellular parasites in host cells.

## Lay abstract

The endoplasmic reticulum (ER) has a central role in maintaining homeostasis and in the regulation of innate immune response. Perturbations in the ER (ER stress) lead to a signaling cascade termed unfolded protein response (UPR), aimed at restoring cell homeostasis. The UPR pathways are strictly connected with innate immunity and inflammation. In fact, many pathogens (mainly viruses and bacteria) are known to induce/modulate the UPR in the host cell. The UPR triggered by intracellular parasites is still poorly investigated. Its characterization could contribute to explain the mechanisms of pathogenicity and to identify targets for the development of new therapeutic approaches.

The endoplasmic reticulum (ER) is involved in several cellular functions, such as synthesis, modification, release and translocation of proteins, lipid and sterols synthesis, metabolism of carbohydrates and calcium storage. A perturbation of the physiological status of the ER (e.g., following an imbalance in the ER folding capacity, nutrient depletion, hypoxia, oxidative stress, disruption of ER calcium ion balance or N-linked glycosylation by drugs such as thapsigargin and tunicamycin) can trigger the ER stress. The response to this stress, named ER stress response or unfolded protein response (UPR), is an elaborate signaling cascade activated to restore ER homeostasis and ensure cell survival. In mammalian cells, three signaling pathways, activated by three ER-transmembrane proteins operating in parallel, mediate the UPR: IRE1, PERK and ATF6. The activation of IRE1 and PERK occurs by oligomerization and autophosphorylation, while ATF6 is translocated to the Golgi apparatus and then activated via proteolytic cleavage.

IRE1 oligomerization activates the C-terminal endoribonuclease domain, which excises 26 base pairs from cytoplasmic XBP1 mRNA, leading to a frame shift that extends the open reading frame and allow the translation of the spliced XBP1 (XBP1s) transcription factor [1]. XBP1s induces the expression of several chaperones, proteins involved in ER-associated degradation (ERAD) system, lipid metabolism [2], proinflammatory cytokines [3] and autophagic response [4]. Under sustained ER stress, IRE1 can also contribute to the degradation of mRNAs that are localized to the ER membrane through a process known as regulated IRE1 dependent decay (RIDD) (Figure 1) [5]. RIDD contributes to maintain ER homeostasis under low ER stress. However, after persistent and unmitigated ER stress RIDD becomes cytotoxic and push the cell toward apoptosis [6]. Moreover, activated IRE1 recruits TRAF2 to the ER membrane to phosphorylate IκB, therefore activating NF-κB and triggering inflammatory pathways [7]. Mammals have two isoforms of IRE1: IRE1α and IRE1β. IRE1α is ubiquitously expressed and its role in UPR signaling has been clearly established. Instead, IRE1β does not cleave XBP1 mRNA and its expression is restricted to intestine and lung, where it may control RIDD [6].

**Figure 1.**
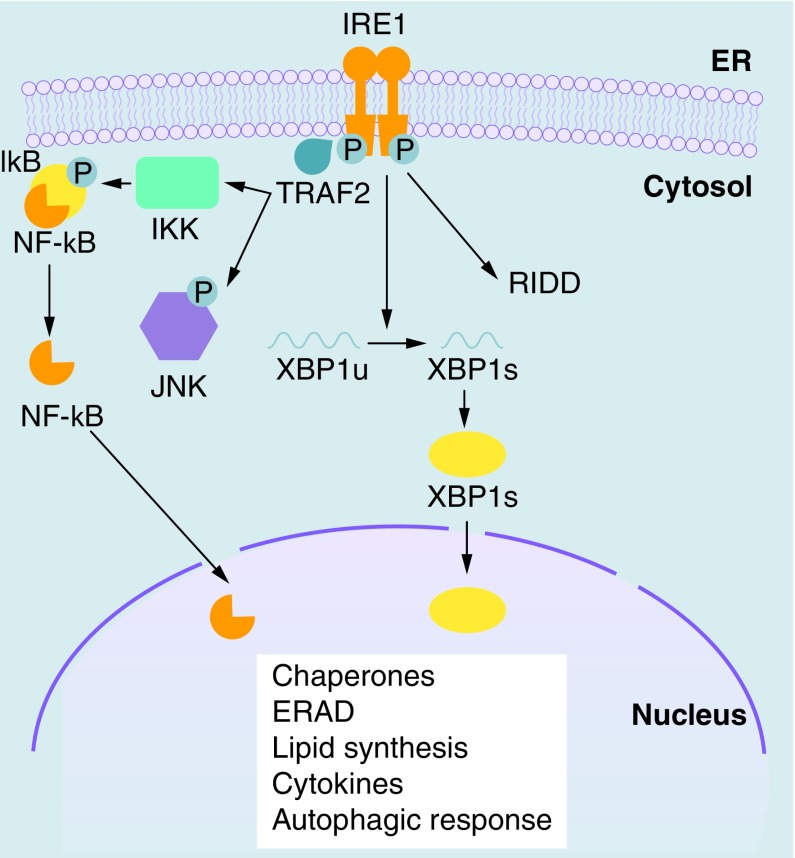
**IRE1 signaling in the unfolded protein response.** IRE1 oligomerization and autophosphorylation activate its endoribonuclease domain, which cleaves XBP1 mRNA, generating an XBP1s mRNA that allows the translation of the active XBP1s transcription factor. IRE1 can also contribute to the degradation of mRNAs associated with ribosomes at the ER through a process known as RIDD. Moreover, phosphorylated IRE1α interacts with IKK and JNK via the recruitment of TRAF2, therefore controlling the activation of the two major inflammatory transcription factors NF-κB and AP-1. ER: Endoplasmic reticulum; IKK: IκB kinase; JNK: c-Jun N-terminal kinase; RIDD: Regulated IRE1-dependent decay.

The activated PERK induces a global translation attenuation by phosphorylation of the α subunit of eIF2α, therefore reducing folding requirements in the ER. Simultaneously, the ATF4 transcription factor escapes inhibition of translation through an alternative translation initiation site. ATF4, in turn, induces the expression of the transcription factor DDIT3/CHOP and GADD34, a phosphatase acting as regulator of eIF2α phosphorylation (Figure 2) [8]. Additionally, PERK induces the expression of genes involved in antioxidant response via phosphorylation of the transcription factor NFE2L2/NRF2 [9].

**Figure 2.**
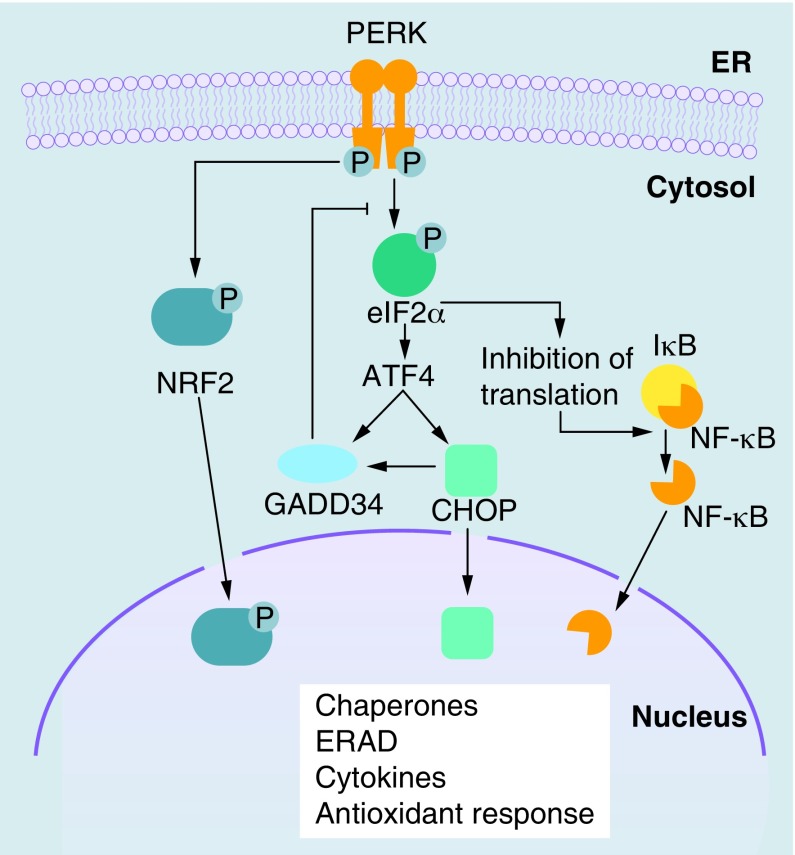
**PERK signaling in the unfolded protein response.** PERK is a transmembrane kinase activated by oligomerization and autophosphorylation. PERK phosphorylates eIF2α, leading to general inhibition of protein translation. The transcription factor ATF4 escapes inhibition of translation through an alternative translation initiation site. ATF4 induces the expression of CHOP and GADD34, a phosphatase acting as regulator of eIF2α phosphorylation. PERK can also phosphorylate and activate the transcription factor NRF2, which induces the expression of genes involved in antioxidant response. PERK-mediated eIF2α phosphorylation and consequent attenuation of translation can promote the activation of NF-κB, since the half-life of its inhibitor (IκB) is much shorter. ERAD: Endoplasmic reticulum-associated degradation; ER: Endoplasmic reticulum.

ATF6 is a transmembrane protein with an N-terminal bZIP transcription factor. Following ER stress, ATF6 translocates to the Golgi apparatus. Here, it is subjected to proteolysis and the transcriptionally active N-terminal fragment is released. The activated ATF6 N-terminal induces the transcription of *XBP1* and contributes to optimization of the UPR by controlling a number of genes related to protein folding and lipid synthesis, some of which are regulated also by XBP1 (Figure 3) [10–12]. Two isoforms of ATF6 have been described: ATF6α and ATF6β, which are both cleaved following ER stress. The ATF6α N-terminal is a strong and rapidly degraded transcriptional activator, whereas ATF6β N-terminal is a weak and slowly degraded transcriptional activator, acting as an endogenous inhibitor of ATF6α [13]. ATF6α and XBP1, by stimulating lipid synthesis, also induce an increase in ER volume, therefore reducing protein–protein aggregation [14].

**Figure 3.**
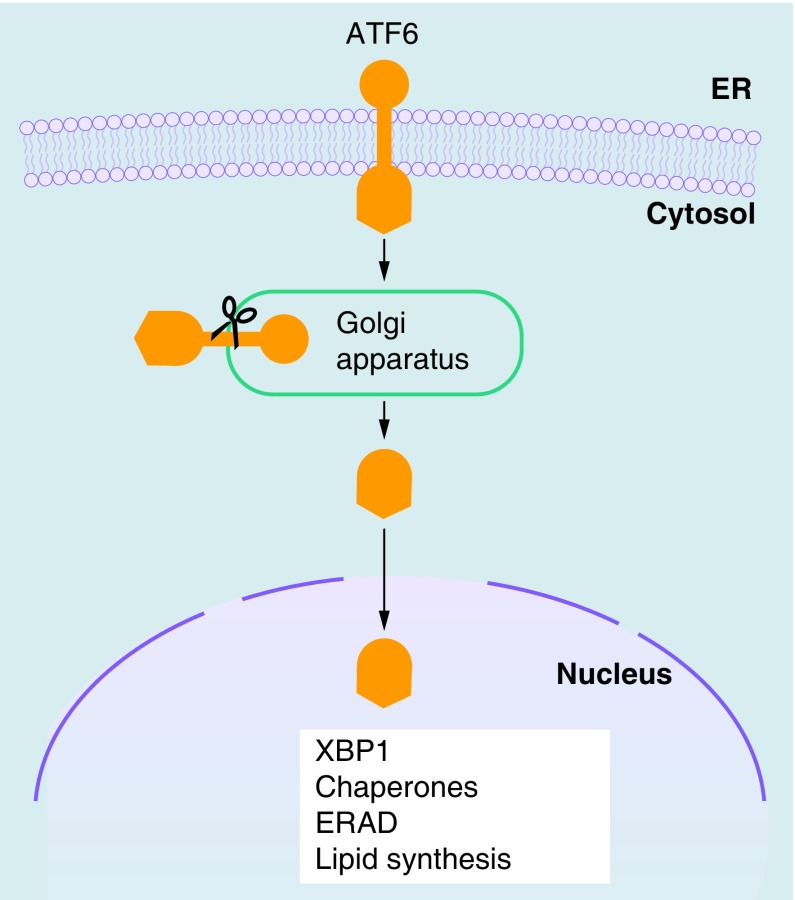
**ATF6 signaling in the unfolded protein response.** ATF6 is localized at the ER in unstressed cells and has a bZIP transcription factor in its cytosolic domain. Following ER stress, ATF6 is transported to the Golgi apparatus, where it is subjected to proteolysis. The cytosolic domain fragment is released and migrates to the nucleus, where it controls the upregulation of XBP1 and genes related to protein folding, ERAD and lipid synthesis. bZIP: Basic leucine zipper; ER: Endoplasmic reticulum; ERAD: Endoplasmic reticulum-associated degradation.

These three signaling pathways have been extensively studied, but communication among them has been less investigated. Currently, it is known that ATF6 induces the transcription of XBP1, and that the increase in IRE1α expression depends on PERK-ATF4 pathway [15]. Together, these three signaling pathways contribute to re-establish the physiological status of the ER reducing the ER stress and ensuring cell survival. Nevertheless, if ER stress is prolonged and cannot be reversed, the cell death occurs, usually by apoptosis and autophagy [16]. The UPR is an evolutionary conserved mechanism across eukaryotes. However, there are differences between metazoans and early-divergent protozoans lacking traditional transcriptional regulation. In fact, protozoans generally do not have recognizable orthologs of IRE1, XBP1 or ATF6, whereas there is evidence for PERK-like control of translation [17].

UPR signaling is traditionally associated with an adaptive response triggered by accumulation of misfolded or unfolded proteins in the ER lumen. In this view, the UPR is aimed at reducing the load of newly synthesized proteins within the ER and eliminate inappropriately folded proteins through upregulation of ER chaperone expression and activation of ERAD pathway. In this context, the ER stress is perceived as a drop of the ER chaperone HSPA5 (known also as GRP78 or BIP), which is engaged by interaction with unfolded proteins, by the sensor domains of IRE1, PERK and ATF6 facing the ER lumen. However, the UPR is not limited to this function and alternative ways to trigger ER stress sensing proteins independently of defects in protein folding exist [18]. Recently, Karali *et al.* [19] showed that VEGF activates IRE1, PERK and ATF6 in endothelial cells through a PLCγ-mediated cross-talk with the mTORC1 complex, independently from accumulation of unfolded proteins in the ER, thereby promoting endothelial cells survival and angiogenesis. Moreover, it was reported that perturbations in the composition of the ER lipid bilayer (i.e., increased membrane lipid saturation) can be sensed by IRE1 and PERK transmembrane domains, independently of changes to protein-folding homeostasis in the ER lumen [20], and that IRE1 and PERK signaling can induce the biosynthesis of fatty acids, phospholipids and cholesterol [21]. Furthermore, yeast IRE1 can be activated by the flavonol quercetin through its binding at the dimer interface of IRE1 [22]. The activation of selected arms of UPR (in particular the IRE1-XBP1 arm) can operate independently of the engagement of the classic UPR involving three signaling pathways. For example, it has been shown that glucose induces IRE1-mediated XBP1 splicing in pancreatic β-cells to expand secretory capacity and increase proinsulin synthesis [23]. The activation of selected branches of UPR is part of the normal differentiation program either in cells that have mainly a secretory function (e.g., pancreas acinar cells, insulin-producing β cells, chondrocytes, osteoclasts, Paneth cells) or in some immune cells [18]. For example, XBP1 has an essential role in differentiation of B cells to plasma cells and in the development and survival of dendritic cells. In particular, in CD8α^+^ dendritic cells XBP1 is constitutively spliced and PERK is also activated [24]. In fully differentiated immune cells, selected UPR pathways have an important role in the regulation of innate immunity and inflammation. In fact, both infections and inflammatory diseases such as atherosclerosis, Type 2 diabetes, cystic fibrosis and inflammatory bowel disease display features characteristic of ER stress (i.e., the induction of classic UPR markers), evidencing a complex cross-talk between UPR, inflammatory and microbial sensing pathways [25].

## The UPR in immunity & inflammation

The UPR is interconnected at different levels with innate immune response pathways, the first line of defense against pathogens. In the innate immune response, pathogen-associated molecular patterns, for example, lipopolysaccharides or nucleic acids such as CpG DNA or dsRNA, are recognized by pattern recognition receptors (PRRs), such as Toll-like receptors (TLRs), nucleotide-binding oligomerization domain (NOD)-like receptors or RIG-I [26] receptors. PRRs activate signaling pathways leading to the expression of genes involved in inflammation, immune cell regulation, survival and proliferation. TLRs are the most characterized PRRs [27]. Interconnections between TLRs and UPR signaling have been described: TLR2 and TLR4 specifically activate the IRE1-XBP1 branch of the UPR, promoting the production of inflammatory mediators (i.e., IL-6) [3]. This activation occurred in the absence of a full ER stress response, demonstrating that a specific arm of the UPR can be activated independently of the others [3].

Recently, Keestra-Gounder *et al.* demonstrated that NOD1 and NOD2, two members of the NOD-like receptor family of PRRs, which are traditionally considered as sensors of bacterial peptidoglycan, have a major role in inducing inflammation during ER stress [28]. The authors showed that the production of the pro-inflammatory cytokine IL-6 was triggered by the ER stress inducers thapsigargin – a specific inhibitor of the sarcoplasmic/endoplasmic reticulum Ca2^+^-ATPase channel – and dithiothreitol (DTT) in a NOD1/2-dependent manner. Moreover, they demonstrated in a murine model that infection with *Brucella abortus*, which is known to induce ER stress [29], triggered inflammation and IL-6 production in a TRAF2, NOD1/2-dependent manner. The pro-inflammatory responses were inhibited by the ER stress inhibitor/chemical chaperone tauroursodeoxycholate (TUDCA) or an IRE1α kinase inhibitor (Kinase-Inhibiting RNase Attenuator 6 [KIRA6]) [30], evidencing a new link between innate immunity and inflammation induced by ER stress.

The activation of NF-κB, a key regulator for immune and inflammatory responses, has being linked to UPR [31,32]. Activation of NF-κB can be promoted through PERK-mediated eIF2α phosphorylation and consequent attenuation of translation (Figure 2). Since the half-life of IκB protein (inhibitor of NF-κB) is much shorter than that of NF-κB, the attenuation of translation reduces NF-κB quenching by neo-synthesized IκB and increase the amount of free NF-κB, independently from IκB phosphorylation [33]. Moreover, the IRE1α–TRAF2 complex can recruit IκB kinase, leading to IκB degradation and the nuclear translocation of NF-κB (Figure 1) [7]. However, NF-κB activation may be dependent from the intensity of UPR. In fact, preconditioning with low dose of ER stress inducers was shown to attenuate NF-κB activation in endothelial cells [34]. Moreover, ER stress can influence NF-κB activity positively or negatively. It has been proposed that NF-κB activation by ER stress occurs in the early phase, whereas its inhibition occurs in the later phase [35]; the inhibition of signaling mediated by NF-κB has shown to be dependent on induction of C/EBP-β by UPR [36].

UPR is also involved in the activation of JNK and in the production of reactive oxygen species (ROS). While JNK can be activated by phosphorylated IRE1α via the recruitment of TRAF2 and ASK1 [37] and by the eIF2α-kinase PKR [38], the production of ROS can occur during the protein folding process, by NADPH oxidase 4 (Nox4), NADPH-P450 reductase and glutathione [39].

## The induction of UPR by pathogens

The UPR pathway is induced in response to a wide variety of cellular perturbations, including nutrient depletion, disruptions of the secretory pathways, accumulation of ROS or increase of free fatty acids. Many of these changes are induced by intracellular pathogens, which subvert the host immune response and cellular processes to establish a compartment that allows their survival and replication. Moreover, many pathogens interact with the ER functions, so it is not surprising that they induce ER stress and UPR [40,41]. In particular, viruses depend on the ER for assembly of virions and budding from cells, therefore perturbing ER homeostasis and causing ER stress. In humans, ER stress response was observed in duodenal biopsies from HIV-infected patients [42] and in livers of patients with chronic HCV infection [43]. The UPR, or selected branches of UPR, can also be triggered by bacteria [44] or some bacterial toxins, such as Shiga toxins [45], cholera toxin [46] and pore-forming toxins. UPR is activated by facultative intracellular bacteria *Brucella melitensis* and *Listeria monocytogenes*. *B. melitensis* extensively interacts with ER during replication, inducing a reorganization of ER around the bacteria and UPR. UPR induction requires both live bacteria and a specific *Brucella* protein [47]. *L. monocytogenes* was found to induce full UPR pathways before entry into host cell. Notably, the *L. monocytogenes* mutant lacking the pore-forming toxin listeriolysin O was unable to induce UPR [48].

Pathogens can trigger a specific branch of the UPR independently of the others, without eliciting full UPR and, in some cases, they appear to actively regulate ER stress signaling. For example, TLR signaling suppresses ATF4-CHOP branch downstream to PERK. TLRs, via the adaptor molecule TRIF, dephosphorylates eIF2B counteracting the inhibitory effects of phosphorylated eIF2α on protein translation, allowing uninterrupted protein synthesis in infected immune cells [49]. Also, TLR stimulation by bacterial ligands in macrophages induces XBP1 splicing but inhibits activation of PERK and ATF6 [3]. Virus mediated UPR activation depends on their infectious life cycle and their immune evasive virulence mechanisms. Viruses would benefit from UPR since increase folding capacity and activation of lipid biosynthesis can sustain viral replication. On the other hand, PERK-mediated inhibition of protein translation, activation of RIDD pathway, the ERAD-mediated degradation of viral proteins and the induction of IFN can have a negative impact on viral replication. Viruses can alter specific branches of UPR to circumvent its detrimental effects. For example, dengue fever virus elicit the ER-signaling pathways depending on timing and the infectious stage, avoiding inhibition of translation, preventing apoptosis and prolonging the viral life cycle [50]. The murine cytomegalovirus protein M50 specifically binds IRE1 and induces its degradation [51]. On the contrary, IRE1 pathway is specifically activated by Japanese encephalitis virus [52]. In this case the beneficial effect of IRE1 was dependent on the activation of RIDD pathway, which led to cleavage of host RNA without showing any effect on Japanese encephalitis virus RNA. Also, influenza A virus infection activates the IRE1 branch of UPR, with little or no activation of the PERK and ATF6 branches. IRE1 activation resulted important for viral replication since its inhibition blocked viral replication [53].

The induction of UPR-related molecules (e.g., ATF4, CHOP, ATF3, GADD34) by microbial products could occur in a PERK independent manner, via TRIF, PKR or GCN2, and does not necessarily reflect a complete UPR. Instead, it could be considered part of a specific transcription program controlled by innate immunity receptors. Therefore, the term ‘microbial stress response’ has been proposed to define these stress pathways [54].

## The UPR in protozoan parasites

ER stress occurs in protozoan parasites during their life cycle, since they are subjected (and need to adapt) to adverse environmental conditions such as nutrient deficiency, hypoxia, oxidative stress, shifts in pH and temperature. Unicellular protozoan parasites, including the causative agents of trypanosomiasis, leishmaniasis, toxoplasmosis and malaria, are able to sense ER stress and organize an UPR, although in a manner different from their host. ER stress response pathways have been investigated in *Trypanosoma brucei* [55,56], *Leishmania* spp. [57], *Toxoplasma gondi* [58] and *Plasmodium falciparum* [59]. These parasites contain a minimal UPR network compared with higher eukaryotic cells [60]. In fact, they lack IRE1 and ATF6, which act along the transcriptional regulatory branches of the UPR. In contrast, an UPR sensor related to PERK is present, which acts on the regulation of protein translation [55,60]. Moreover, in *T. brucei*, a post-transcriptional program called spliced leader silencing (SLS) pathway is elicited upon ER stress. The activation of SLS pathway causes major reduction of mRNAs with consequent inhibition of protein synthesis and activation of a programmed cell death pathway. It has been hypothesized that SLS pathway could be used by the parasites as an analog to apoptosis observed in higher eukaryotes to rapidly eliminate unfit organisms from the population [55].

## The UPR in parasitized cells

A considerable amount of work has been done in the last years to characterize the role and modulation of UPR pathways in cells infected by viruses and bacteria. On the contrary, the study of UPR pathways in cells infected by intracellular protozoan parasites can be still considered in its infancy. For instance, the UPR pathways have been investigated in infection by Apicomplexan and Trypanosomatid protozoan parasites, including the causative agents of malaria, toxoplasmosis, cryptosporidiosis and leishmaniasis (Table 1).

**Table 1.** **Protozoan parasites that affect the host unfolded protein response.**

**Parasite**	**Infection model**	**Affected host UPR branch/component**	**Ref.**
*Plasmodium berghei*	*In vitro* (Hepa 1–6 cells), *In vivo* (C57BL/6 mice; liver)	IRE1-XBP1, PERK, ATF6	[61]
	*In vivo* (C57BL/6 mice; brain)	IRE1-XBP1, PERK, ATF6	[62]
*Toxoplasma gondii*	*In vivo* (BALB/c mice), *In vitro* (293T, HFFs cells)	ATF6	[63]
	*In vitro* (murine neural stem cells)	CHOP	[64]
	*In vitro* (C17.2 neural cells)	CHOP	[65]
*Cryptosporidium parvum*	*In vitro* (HCT-8 cells)	PERK/NRF2	[66]
*Leishmania amazonensis*	*In vitro* (RAW 264.7 cells; murine primary macrophages)	IRE1-XBP1	[67]
*Leishmania infantum*	*In vitro* (U937 cells; murine primary macrophages)	XBP1, PERK-ATF4	[68]

UPR: Unfolded protein response.

### Plasmodium berghei

*Plasmodium* spp, the etiological agents of malaria, are obligate intracellular apicomplexan parasites. In mammals, the motile sporozoites infect hepatocytes, develop into merozoites, which are released in the bloodstream and invade red blood cells, leading to disease. The endothelial dysfunction and tissue inflammation contribute to several malaria complications (i.e., acute respiratory distress, cerebral malaria or placental malaria) [69].

The role of UPR in hepatocytes infected with *P. berghei* has recently been investigated in *in vitro* and *in vivo* murine models [61]. Mostly in the liver, the UPR is interconnected with metabolic pathways such as lipid and glucose metabolism [70]. Importantly, a further hepatocyte-specific UPR branch exists, mediated by the ER transcription factor CREBH, which does not activate protein folding transcriptional programs but rather regulates liver metabolic pathways [71,72]. It has been reported that *P. berghei* induces UPR in hepatocytes via both XBP1 and CREBH pathways. Moreover, also PERK and ATF6 branches appear to be involved. The elimination of XBP1 splicing or knockdown of *CREBH* is detrimental to parasite development, indicating a beneficial role of the host UPR for *Plasmodium* in hepatocytes infection [61]. It is possible that UPR supports parasite growth by regulating lipid metabolism, particularly that of phosphatidylcholine. In fact, XBP1s can induce the synthesis of phospholipids, such as phosphatidylcholine [73], which is necessary for the correct localization of parasite proteins to the membrane of parasitophorous vacuole (PV) and it is essential for parasite survival during liver stage infection [74].

Cerebral malaria is one of the most serious complications of *Plasmodium* infection. An experimental murine model of cerebral malaria, induced by the infection of susceptible mice with *Plasmodium berghei*, has been used to examine the role of ER stress response in modulating neuronal cell death induced by this parasite [62]. The brains of infected and uninfected mice were analyzed by western blotting and immunohistochemistry, showing the activation of the three ER stress sensors ATF6, PERK and IRE1α. Moreover, p-eIF2α, XBP1s, CHOP, ATF4, GADD34 were also significantly upregulated, accounting for a complete UPR activation in this infection model. The association of these results with monitoring of apoptotic markers indicated a role of UPR in modulating neuronal cell death in this experimental cerebral malaria model.

### Toxoplasma gondii

*Toxoplasma gondii* is an obligate intracellular parasite belonging to the phylum Apicomplexa. This parasite can infect any cell type, causing severe disease in immunocompromised individuals. It also causes abortion, and cognitive defects in newborns. During cell invasion *T. gondii* secretes numerous proteins directed to the host cell nucleus or to the surface of PV. ROP18 is a Ser/Thr protein kinase that is secreted into the host cell, where it associates to the surface of the PV membrane [75]. Recently, ATF6β, which act as transcription factor in the UPR pathway during ER stress and resides in the host ER, was identified as a ROP18 target [63]. The phosphorylation of ATF6β by ROP18 induced its proteosomal degradation and reduction in ATF6β-mediated gene expression after induction of UPR. ATF6β-deficient mice exhibit a high susceptibility to infection by the parasite, indicating that ATF6β has a key role in resistance against *T. gondii* infection [63].

The mechanism of neuropathogenesis in brain toxoplasmosis has been investigated in murine neural stem cells isolated from mouse embryos [64] and in C17.2 cells [65]. The authors found that *T. gondii* infection induced apoptosis in murine neural stem cells through activation of CHOP, caspase-12 and JNK, which are associated with UPR [64,65].

### Cryptosporidium parvum

*Cryptosporidium parvum* is an intracellular parasite of both human and veterinary interest, belonging to phylum Apicomplexa, class Coccidia. *C. parvum* is more closely related to *Plasmodium* spp. than other Coccidia. Both *C. parvum* and *T. gondii* are dependent upon host-derived polyamines [76]. In particular, *C. parvum* lacks ornithine decarboxylase. Polyamines are charged molecules and their transport across cell membranes in the absence of energy transporters requires neutralization of their charge by acetylation. The intracellular *C. parvum* is separated from the host cell cytoplasm by two sets of membrane bilayers. Morada *et al.* [66] recently showed that infection of human epithelial HCT-8 cells by *C. parvum* results in elevated activity of host SAT1 in the infected cells and increase in intracellular acetylspermine, which can be taken up by the parasite. The authors also found the increase of several UPR markers in infected HCT-8 cells, including phosphorylated eIF2α, CHOP, NRF2 and the UPR-related chaperones GRP78 and calreticulin. Taken these results together, and since NRF2 contributes to the expression of SAT [77], the authors hypothesized that invasion of HCT-8 cells by *C. parvum* can induce UPR that leads to increase of host cell SAT1 and N1-acetylpolyamines, which can be used by a parasite that lacks ornithine decarboxylase.

### *Leishmania* spp

Leishmaniases are vector-borne diseases caused by the obligate intracellular parasites belonging to the genus *Leishmania* (Trypanosomatidae). Leishmaniases are endemic in 98 countries. It has been estimated that 0.2–0.4 million cases of visceral leishmaniasis and 0.7–1.2 million cases of cutaneous leishmaniasis occur each year, causing 20,000–40,000 deaths per year [78]. *Leishmania* has evolved complex strategies to establish infection and survive within macrophages, counteracting macrophage defenses such as oxidative damage, immune activation, antigen presentation and apoptosis, at the same time improving nutrient availability [79]. Recently, the cellular responses induced by infection with *Leishmania major* in macrophages from resistant C57BL/6 mice has been investigated, evidencing an inflammatory response, mediated by ROS and JNK signaling, triggered by a stress stimulus provided by the parasite [80]. However, the role of UPR in infected cells remains poorly investigated.

The role of UPR during *Leishmania* infection has been investigated in RAW 264.7 macrophages infected with *L. amazonensis* [67]. It has been shown that *L. amazonensis* infection activates the IRE1-XBP1 arm of UPR in host cells in a TLR2-dependent manner, leading to the expression of IFN-β, which has an established role in *L. amazonensis* pathogenesis [81]. Moreover, XBP1s was necessary to sustain the expression of the antioxidant gene HO-1, that inhibited ROS production. The authors concluded that the activation of XBP1 has an important role in infection by increasing IFN-β expression and protecting the parasites from oxidative stress, thereby promoting parasite proliferation [67].

More recently, we showed that *L. infantum* infection induce a mild UPR in U937-derived macrophages [68], confirming – although with some differences – the activation of IRE1-XBP1 arm of UPR observed in infection by *L. amazonensis*. Moreover, a potential involvement of the PERK–ATF4 branch of UPR during *Leishmania* infection was also evidenced since *ATF4*, as well as downstream genes such as *ATF3* and *CHOP* were significantly upregulated [68]. It is known that the PERK/eIF2α/ATF4 pathway plays a key role in autophagy regulation. In fact, the transcription of *MAP1LC3B*, an essential autophagy gene, can be induced by ER stress through the activity of ATF4 [82]. Moreover, ER stress can promote autophagy through induction of DDIT4/REDD1, an inhibitor of mTOR [83], in an ATF4-dependent manner [84]. Cyrino *et al.* showed that *L. amazonensis* induces autophagy in macrophages and that its inhibition with 3-methyladenine reduced the infection index, suggesting that the autophagic process could provide nutrition to the parasite [85]. We found that *MAP1LC3B* was significantly induced in U937-derived macrophages infected by *L. infantum* [68]. Therefore, it is likely that also the PERK–ATF4 branch of the UPR could have a role in *Leishmania* infection (e.g., induction of autophagic process). However, this aspect will need further investigations.

It is known that low levels of ER stress may be beneficial to cells by eliciting a mild (adaptive) UPR, which can increase the cellular resistance to subsequent ER stress (ER hormesis) [86]. We found that the effects of ER stress inducers tunicamycin and DTT (i.e., eIF2α phosphorylation and CHOP protein induction) were attenuated/delayed in U937 and THP1-derived macrophages infected by *L. infantum*, accounting for a protective role of host UPR at least in the initial phase of infection [68]. This can further point out the role of IRE1-XBP1 and PERK-ATF4 arms of the UPR in *Leishmania* infection, since both these arms contribute to the ER hormesis. Establishing if the parasite can actively modulate the host UPR, curbing it to a mild response, will need further investigations.

## Conclusion

The UPR is deeply interconnected with inflammation and immunity. In fact, the UPR pathways can be triggered and/or modulated not only by accumulation of misfolded or unfolded proteins in the ER lumen but also by pathogen infections and inflammatory conditions. Moreover, the UPR can promote the induction of several cytokines, including type I IFN. Despite the UPR has been studied in bacterial and viral infections, the induction/modulation of UPR pathways in cells infected by intracellular protozoan parasites is still poorly investigated. Few recent works indicate that host UPR can have a role in establishing intracellular parasite infection. In fact, it has been shown that the host UPR induction or modulation by the parasites belonging to genus *Plasmodium*, *Leishmania*, *Toxoplasma* and *Cryptosporidium* can have a role in the pathogenesis and/or favor the infection. However, much more research is needed to understand the complex interactions between host UPR signaling pathways and the protozoan parasites, and to exploit this knowledge to design new drugs.

## Future perspective

It will be important to deeply characterize at the molecular level the UPR pathways triggered by protozoan parasite infection and to understand how these pathways intersect with other stress responses such as autophagy and oxidative stress response. Since the ER is pivotal in detecting cellular insults and triggering specific responses, the understanding of the UPR in intracellular parasite infections may explain some of the strategies that the parasites have evolved to survive/replicate into host cell. As the knowledge regarding interaction between parasites and cellular stress responses (in particular UPR) will grow, new targets for the development of drugs targeting ER stress or specific branches of UPR could be revealed. This could lead to the development of more effective/new therapeutic approaches, particularly useful in the cases of pharmacological resistance or toxicity of existing therapies. Moreover, the differences in UPR network between host and parasites could be exploited. For instance, the fact that *L. donovani* was more sensitive to DTT treatment than host macrophages [60] indicates that molecules inducing UPR may be used to selectively target the parasite.

**Executive summary**

- The unfolded protein response (UPR) is a signaling cascade activated to restore endoplasmic reticulum (ER) homeostasis and ensure cell survival following an imbalance in the ER folding capacity. The UPR is mediated by three ER-resident transmembrane proteins: IRE1, PERK and ATF6.
- The UPR can also be part of the normal differentiation program in some immune cells and in cells that have a secretory function.
- The UPR is interconnected with innate immune response pathways and inflammation via pattern recognition receptors, reactive oxygen species production and activation of c-Jun N-terminal kinase and NF-κB.
- Many intracellular pathogens, particularly viruses and bacteria, are known to induce an UPR response in the infected cell. Pathogens can trigger a specific branch of the UPR independently of the others, without eliciting full UPR. In some case, they appear to actively regulate ER stress signaling.
- The knowledge about UPR pathways in cells infected by intracellular protozoan parasites is still limited. In the last years, these pathways have started to be investigated in infection by parasites belonging to genus *Plasmodium*, *Leishmania*, *Toxoplasma* and *Cryptosporidium*.
- It is emerging that the host UPR induction or modulation by these parasites can have a role in the pathogenesis and/or favors their infection. However, much more research is needed to understand these complex interactions.
- The molecular characterization of the UPR pathways triggered by parasite infection will allow to better understand how these pathways intersect with other stress responses (e.g., autophagy and oxidative stress response) and, since the ER is pivotal in detecting cellular insults, it could explain some of the strategies that the parasites have evolved to survive/replicate into host cell.
- Finally, further characterization of UPR pathways induction/modulation by intracellular parasites could allow the identification of novel therapeutic targets for the development of new drugs.
